# Factors associated with the severity of road traffic injuries from emergency department based surveillance system in two Mexican cities

**DOI:** 10.1186/s12873-022-00576-x

**Published:** 2022-02-04

**Authors:** Lourdes Gómez-García, Elisa Hidalgo-Solórzano, Ricardo Pérez-Núñez, Vanessa F. Jacobo-Zepeda, Ricardo G. Ascencio-Tene, Jeffrey C. Lunnen, Amber Mehmood

**Affiliations:** 1 Center for Health Systems Research, National Institute of Public Health, Universidad #655, Colonia Santa María Ahuacatitlán, Cerr los Pinos y Caminera, CP 62100 Cuernavaca, Morelos Mexico; 2 Hospital General de León, León, Guanajuato Mexico; 3 Antiguo Hospital Civil de Guadalajara “Fray Antonio Alcalde”, Guadalajara, Jalisco Mexico; 4 Johns Hopkins International Injury Research Unit, Johns Hopkins Bloomberg School of Public Health, Baltimore, MD USA; 5 University of South Florida College of Public Health, Tampa, FL USA

**Keywords:** Road traffic injuries, Surveillance system, Injury severity score, Mexico

## Abstract

**Background:**

Limited data from low- and middle-income countries (LMICs) on the severity of road traffic injuries (RTIs) and their relation to different variables of interest are routinely obtained. Knowledge on this subject relies on evidence from high-income countries, which might not be the same as in LMICs. This information is greatly needed to advance and inform local and regional efforts towards the United Nations’ Decade of Action and the Sustainable Development Goals.

**Methods:**

From May 2012 to November 2014, a RTI surveillance system was implemented in two referral hospitals in two Mexican cities, León and Guadalajara, with the objective of exploring the relationship between Injury Severity Score (ISS) and different sociodemographic characteristics of the injured as well as different variables related to the event and the environment. All individuals suffering RTIs who visited the Emergency Rooms (ER) were included after granting informed consent. A Zero-Truncated Negative Binomial Model was employed to explore the statistical association between ISS and variables of interest.

**Results:**

3024 individuals participated in the study: 2185 (72.3%) patients from León and 839 patients (27.7%) from Guadalajara. Being male, in the 20–59 age-group, having less schooling, events occurring in Guadalajara, on Sundays, at night, and arriving at ER via public/private ambulance were all associated with an increased log count of ISS. Found a significant interaction effect (*p*-value< 0.05) between type of road user and alcohol intake six hours before the accident on severity of the injury (ISS). The use of illicit drugs, cellphones and safety devices during the event showed no association to ISS.

**Conclusions:**

Our study contributes to the statistical analysis of ISS obtained through RTI hospital surveillance systems. Findings might facilitate the development and evaluation of focused interventions to reduce RTIs in vulnerable users, to enhance ER services and prehospital care, and to reduce drink driving.

## Background

Road traffic injuries (RTIs) remain a problem worldwide. In 2016, RTIs caused 1.3 million deaths, representing the eighth leading cause of death globally, and more than 50 million injuries, with many experiencing a permanent disability as a result of their injury [1]. More than 90% of road traffic deaths occur in low- and middle-income countries (LMICs), and road traffic injuries affect people from lower socioeconomic levels even in high-income countries (HICs) [1].

Evidence has been generated on the influence of diverse factors on the occurrence of road traffic crashes and the severity of the injuries sustained. However, road safety relies upon the conjunction of various components such as human factors, protective devices, traffic flow, road infrastructure, and environmental conditions that may also influence the severity of the resulting injuries [2, 3]. On the other hand, since severe injuries are associated with higher mortality, extended hospitalization and significant economic implications at both the level of health services as well as the individual, [4] this information is also useful to establish resources needed to be assigned to secondary and tertiary injury prevention. Costs may differ by socioeconomic status and road user category as well. A previous study documented that pedestrians involved in a road traffic crash have to pay higher health care costs than people injured in crashes between cars, 80% of pedestrians involved in a crash incurred out-of-pocket expenses in comparison to only 45% of car crash victims [5]. Despite their utility, limited data from LMICs are routinely collected on the severity of RTIs, making it difficult to evaluate their relation to different variables of interest in these contexts.

Between 2018 and 2019, more than 1.3 million people in Mexico suffered non-fatal RTIs, with almost 22% reporting permanent health consequences, contributing to the burden of disability due to non-intentional injuries [6]. Additionally RTIs are one of the leading causes of potential years of life lost within the country, especially among men [7]. RTIs also impose a significant burden on Mexican society: economic [8, 9] and intangible costs significantly impact both the injured person and their households [10]. In Mexico, few studies have examined the severity of RTIs and their relationship with mortality or morbidity in the past. Among the most important efforts, Hidalgo-Solórzano et al. (2005) collected information on all injured patients admitted to three hospitals in Cuernavaca for three months. A total of 376 individuals were analyzed, of which 66% were RTIs [11]. Ávila-Burgos L et al. (2012) collected information on 8920 injured patients from seven hospitals distributed within three Mexican cities during one month. Of these, 13.6% were RTIs [12]. Evidence from these two studies did not include an analysis of the specific type of road user or exposure to different risk and protective factors—including key environmental characteristics of the event. In both studies injury severity was measured using only the most severe injury’s Abbreviated Injury Score (AIS) and was analyzed through a bivariate regression analysis comparing “severe” (AIS-score 1–2) versus “not severe” (AIS-score 3–6) patients. This did not account for the effect of severity from multiple injuries, which is crucial for a correct analysis of this variable [13]. Finally, both samples were collected during short periods, which yielded small sample sizes for the cities under study.

To overcome these methodological limitations, as part of *Global Road Safety Program* monitoring and evaluation activities in Mexico, the National Institute of Public Health of Mexico and the Johns Hopkins International Injury Research Unit implemented a hospital-based injury surveillance in two large public sector hospitals during 2012–2014. In coordination with local authorities, these hospitals were identified as the major referral centers for RTIs, and included the General Regional Hospital of León in Guanajuato, and the Civil Hospital Fray Antonio Alcalde in Guadalajara, Jalisco. Evidence has shown that hospital-based injury surveillance systems are a useful method to obtain risk factor information and to evaluate the effectiveness of interventions [14]. Hospital-based surveillance data allows for detailed information that is useful for characterizing severity, outcomes and the burden of injuries on public health [15].

To advance national efforts towards the accomplishment of the 2011–2020 Decade of Action for Road Safety and the Sustainable Developments Goals promoted by the United Nations, [16, 17] the objective of the present study was to analyze the association of RTI severity with sociodemographic, the event and environmental variables. This information would be useful to strengthen local, regional and LMICs efforts.

## Methods

### Study design and sample selection

This cross-sectional study analyzes primary data from an emergency department based RTI surveillance system in two Mexican cities. Information was collected in León from May 16th to November 15th, 2012, from April to December 2013, and January to November 2014. Information was collected in Guadalajara from July to December 2013 and from May to November 2014. The study period differed between hospitals due to administrative changes in both facilities. The hospital in León is a second-level hospital with 221 hospital beds while the hospital in Guadalajara is a third-level hospital with 843 beds. Both hospitals are publicly funded.

All individuals suffering RTIs who visited the Emergency Rooms (ER) or that were hospitalized in the selected hospitals during the study period were eligible for inclusion, after obtaining oral informed consent. When the injured person was not able to consent, a relative or guardian provided such information. People who died on site or during emergency transportation to the hospital, and people who did not agree to participate were excluded of the study. The non-response rate was 4.5% (*n* = 136).

The data collectors were trained in data abstraction, injury surveillance guidelines and ethical research conduct. The data collection team recorded data on a standardized questionnaire from three different sources: directly from the patient (or relatives), from paramedics or hospital staff, and the patients’ medical records. The data collection process covered each person from hospital admission until discharge, and included the patient’s sociodemographic information, details of different injuries sustained, exposure to risk and protective factors associated with road traffic collisions, and contextual information about the collision. Study supervisors collected the forms and entered all data via Microsoft Visual FoxPro 9.0.

### Measures

A detailed injury description was obtained through medical charts and was categorized using a matrix, which related the nature of the injury sustained and the part of the body that was affected. All injuries in the matrix were classified according to the Abbreviated Injury Scale 2005 Update 2008 (AIS) [18]. This scale is a guide to measure if an injury may be a threat to life. For this purpose, the AIS offers an ordinal scale that ranges from the least severe (AIS = 1) to the most severe and currently untreatable injury (AIS = 6) [13, 18]. To analyze the severity of multiple injuries sustained, we calculated the Injury Severity Score (ISS), [13] which is the sum of the squares of the highest AIS scores in three out of six different body regions [18, 19].

Due to the characteristics of this matrix, different AIS codes will match with the general injury description. For example, if the injury matrix describes an affected organ with no bleeding in the neck, we can presume that the organ affected may be the esophagus, larynx, pharynx, salivary gland, thyroid gland, trachea or vocal cord. We considered any possibility between the limits of the minor severity (no internal bleeding) and we used a conservative approach, in accordance with the AIS coding recommendations [18]. We considered in advance that some of the most severe injuries might be underestimated through this method, so we decided to include additional information from the emergency room diagnosis to triangulate and validate information obtained from the matrix.

Sociodemographic variables from the injured included sex, age, medical insurance status, and schooling. For the final model, schooling was categorized as 0 when individuals had less than six years of schooling or schooling was not specified (as there were no statistical differences among these two groups); 1 when individuals finished six to eight years of schooling; 2 for those with nine to eleven years of schooling; and 3 for individuals with 12 or more years of schooling. To categorize type of road user, we employed International Statistical Classification of Diseases and Related Health Problems (ICD-10) criteria as follows:

•Pedestrians: V02–V04 (.1, .9), V09 (.2, .3, .9);

•Cyclists: V12–V14 (.3–.9), V19.4–V19.6;

•Motorcyclists: V20–V28 (.3–.9), V29–V39 (.4–.9);

•Four-wheeled vehicle occupant (car, pick-up truck, heavy transport or bus occupants): V40–V79 (.4–.9).

To document the exposure to alcohol six hours before the event, helmet use and the use of restraint devices (seatbelt and child restraint systems), we relied on three sources of information: the patient’s self-report, the medical examination and the official medical record. We constructed a summary variable that included the information from all sources. If any source had an affirmative response, we assumed exposure to these risk and or protective factors. We generated a variable to capture the effect of using safety devices (helmets for both cyclists and motorcyclists, and seatbelt or child restraints systems for four-wheelers). As this information was not explored for pedestrians, they were assumed not to be using any safety device, which might have not been the case (i.e. use of helmet by roller-skaters or skateboarders). The use of a cellphone while driving a four-wheel vehicle, riding both bicycles and motorcycles or walking was documented only through self-report.

Environmental variables included day of the week, presence of daylight (any event reported between 07:00 h and 19:00 h), the municipality where the event occurred, means of transportation to ER (0: using their own resources, 1: using public/private sector service, and 2: not specified), and the duration of time before receiving medical attention in minutes.

### Data analysis

The analysis was conducted using STATA 14®. A univariate analysis reported summary measures for each variable in order to describe the main characteristics of the patients, the exposure to different risk factors and environmental factors from the context and the event itself. To analyze factors associated with ISS, we considered the nature of this variable. In theory, ISS is expressed as a count data with 0 as the lowest limit (no injury at all) and 75 as the highest (representing either at least one injury with severity AIS-2008 code 6 or “maximal” –currently untreatable–, or the sum of the squares of three critical injuries). Since our sample included only injured individuals, the minimum observed was 1 (the minimum value possible for a minor injury).

There are different alternatives to help overcome the methodological challenges associated with analyzing these types of variables. Using a statistical method that is more appropriate for the type of data collected is one of them [20]. Zero-Truncated Negative Binomial regression has been proven to be useful to model count data for which the value zero cannot occur and when there is evidence of over dispersion [21, 22]. As these two characteristics were present in the ISS, we adjusted a Zero-Truncated Negative Binomial regression saturated model including all variables found to be associated during the bivariate analysis with a *P*-value < 0.25 to control for potential confusion [23, 24]. We tested several interactions of interest such as the interaction of age and sex, alcohol use and exposure to other risk factors (nonuse of safety devices such as a seatbelt or helmet, use of cellphone), day/time of occurrence and use of alcohol, type of road user and alcohol use, and daylight with day of occurrence. To obtain the most parsimonious model, we followed a backward elimination approach; the final model was selected based on the Akaike’s Information Criteria. After the Zero-Truncated Negative Binomial model, we estimated values of ISS for different profiles, calculated from predictions of model. Using the *margins* command, this estimation were fit at fixed values of some covariates (sex, age, road user, alcohol consumption, day of week, time of day and city) and averaging over the remaining covariates. We also estimated 95% confidence intervals.

## Results

### Descriptive analysis

From the 3034 injured patients included, complete information was obtained for 3024 individuals (99.67%). The majority was from León (72.3%), males (76.2%) and in the 20–59 age group (65.0%). The mean age was 29.9 with a standard deviation (SD) of 15.5 and a range of 0–99 years. As shown in Table 1, 21.2% did not complete six years of schooling, 19.8% completed between the six and eight years of schooling, 38.9% completed between nine and eleven years of schooling, and 17.4% completed twelve or more years of schooling. In terms of health insurance, 69.8% of participants reported to be insured; 95.7% of those by “Seguro Popular,” a public health care insurance scheme designed for those not insured by other social security institutions. Motorcyclists (29.9%) were the most affected road user, closely followed by four-wheeled and other vehicle occupants (29.5%); pedestrians (21.0%), and cyclists (19.6%).

**Table 1:** Demographic characteristics of road traffic injured from León and Guadalajara, Mexico

Variables		Guadalajara	León	Total
		***N*** = 839	***N*** = 2185	***N*** = 3024
		%	%	%
**Sex**^a^	Female	23.6	23.4	23.5
	Male	75.9	76.3	76.2
	Not specified	0.5	0.3	0.3
**Age group**^b^	Under 10	0.4	6.0	4.4
	10-19	23.0	25.3	24.6
	20–34	42.7	38.5	39.7
	35–59	28.1	24.3	25.4
	60 years and older	5.6	5.7	5.7
	Not specified	0.2	0.3	0.3
**Medical Insurance**^b^	Uninsured	18.7	32.5	28.7
	Insured	80.7	65.7	69.8
	Not specified	0.6	1.8	1.5
**Schooling**^b^	Less than 6 years	16.7	22.9	21.2
	6–8 years	17.4	20.8	19.8
	9–11 years	36.1	39.9	38.9
	12 and more years	27.2	13.7	17.4
	Not specified	2.6	2.8	2.7
**Road user**^b^	Pedestrians	15.5	23.1	21.0
	Cyclists	11.9	22.6	19.6
	Motorcyclists	39.0	26.4	29.9
	Four-wheeled & other vehicle occupants	33.6	27.9	29.5

^a^ *p*-value ≥0.05; ^b^ *p*-value < 0.001

Exposure to risk factors was common: 12.3% of all road users had consumed alcohol six hours before the traffic collision; 96.8% of cyclists were not wearing a helmet as compared to 44.7% of motorcyclists; 93.2% of children and 69.1% of adults traveling in a four-wheeler were not using child restraints or seatbelts, respectively (Table 2).

**Table 2:** Exposure to risk factors present in road traffic injured from León and Guadalajara, Mexico

Variables		Guadalajara	León	Total
		N = 839	N = 2185	***N*** = 3024
		%	%	%
**Alcohol consumption**^a^	Yes	22.9	8.3	12.3
	No	76.2	90.5	86.5
	Not specified	1.0	1.2	1.1
**Use of illicit drugs**^a^	Yes	2.3	1.1	1.4
	No	92.4	97.4	96.0
	Not specified	5.4	1.6	2.6
Use of safety devices^*a^	Yes	20.3	26.8	25.0
	No	57.5	45.6	48.9
	Not specified	22.3	27.6	26.1
**Cellphone use**^a^	Yes	2.7	0.4	1.0
	No	88.4	97.7	95.1
	Not specified	8.8	1.9	3.8
**Work related**^a^	Yes	15.6	18.5	17.7
	No	70.4	78.5	76.3
	Not specified	14.0	2.9	6.0

^*^ Helmet use in cyclists and motorcyclists and seatbelt use in four-wheeled vehicle occupants; ^a^ *p*-value < 0.001

The environmental characteristics of the road traffic events are described in Table 3. Around one-third of the events occurred at night (34.8%) and on weekends (30.2%). Interestingly 52.8% arrived at the hospital by their own means. Excluding sex, the distribution of variables was statistically different between the two cities.

**Table 3:** Environmental factors in road traffic injuries from León and Guadalajara, Mexico

Variables		Guadalajara	León	Total
		N = 839	N = 2185	N = 3024
		%	%	%
**Day of the week**^a^	Monday	14.8	16.6	16.1
	Tuesday	14.9	13.1	13.6
	Wednesday	12.4	14.1	13.6
	Thursday	13.5	13.1	13.2
	Friday	9.5	14.7	13.2
	Saturday	13.8	13.4	13.5
	Sunday	21.1	15.0	16.7
**Time of the day**^a^	Nighttime (20–06 h)	40.5	32.6	34.8
	Daylight (07–19 h)	59.5	67.4	65.2
**Municipality where the event occurred**^a^	Same Metropolitan Area	61.6	82.9	77.0
	Different	29.0	9.4	14.9
	Not specified	9.4	7.6	8.1
**Means of transportation to ER**^a^	On their own	61.9	49.4	52.8
	Public/private service	37.7	50.2	46.7
	Not specified	0.5	0.5	0.5
**Time in minutes before receiving medical attention**^a^	30 min or less	54.4	37.5	42.2
	31–60 min	22.7	23.9	23.5
	61 min or more	20.1	37.0	32.3
	Not specified	2.9	1.7	2.0

^a^ *p*-value < 0.001

The time before first receiving medical attention ranged from 2 min to 21,600 min (15 days), with a median value of 40 min and interquartile range (IQR) = 150. Around 42.2% of the sample received medical care within 30 min or less, 23.5% between 31 to 60 min and 32.3% after one hour. ISS in our sample ranged from 1 to 59 and had a mean value of 5.6 (±5.0) and a median of 4.5.

Table 4 shows different factors associated with the ISS. As can be seen, the log count of ISS for males was 0.19 greater than for females, and of those in the 20–59 age-group was 0.17 greater than for children. Schooling showed a gradient of a negative association with the log count of ISS: the higher the schooling, the lower the log count of ISS. Compared with four-wheeled and other vehicle occupants that had not consumed alcohol, four-wheeled and other vehicle occupants who consumed alcohol and pedestrians that reported not having consumed alcohol had a higher log count of ISS. However, pedestrians and cyclists that had consumed alcohol had a lower log count of ISS than four-wheeled and other vehicle occupants that had not consumed alcohol. The log count of ISS was positively associated with events occurring on Sundays, at nighttime, and arriving at ER via public or private ambulance services. Patients recruited in Guadalajara had 0.391 higher log count of ISS than those recruited in León.

**Table 4:** Factors associated to Injury Severity Score in persons suffering RTIs from Guadalajara and León, Mexico

Variables	Crude Coefficient		95% CI			Adjusted Coefficient^a^		95% CI		
Male	0.242	***	0.158	,	0.326	0.185	***	0.113	,	0.257
Sex not specified	0.527		−0.063	,	1.117	0.285		−0.175	,	0.746
10–19 age group	0.123		−0.062	,	0.308	0.131		−0.032	,	0.293
20–59 age group	0.221	*	0.045	,	0.396	0.167	*	0.013	,	0.320
60 years and older	0.324	**	0.100	,	0.548	0.148		−0.032	,	0.328
Age not specified	0.532		−0.148	,	1.212	0.112		−0.416	,	0.640
Insured	0.052		−0.026	,	0.130	0.013		−0.050	,	0.077
Insurance status not specified	0.553	***	0.272	,	0.834	0.303	**	0.084	,	0.522
6–8 years of schooling	−0.132	*	−0.238	,	−0.026	− 0.109	*	− 0.197	,	−0.020
9–11 years of schooling	− 0.194	***	− 0.284	,	− 0.103	− 0.151	***	− 0.231	,	− 0.071
12 and more years of schooling	− 0.190	**	− 0.300	,	− 0.080	− 0.149	**	− 0.245	,	− 0.054
Pedestrians that hadn’t consumed alcohol	0.142	**	0.039	,	0.244	0.135	**	0.048	,	0.222
Cyclists that hadn’t consumed alcohol	−0.294	***	− 0.401	,	− 0.186	− 0.090		− 0.184	,	0.005
Motorcyclists that hadn’t consumed alcohol	−0.010		− 0.106	,	0.087	−0.024		− 0.107	,	0.060
Four-wheeled and other vehicle occupants that consumed alcohol	0.624	***	0.462	,	0.786	0.231	**	0.096	,	0.366
Pedestrians that consumed alcohol	−0.357	**	−0.622	,	−0.092	−0.218	*	−0.433	,	−0.003
Cyclists that consumed alcohol	−0.509	**	−0.832	,	−0.185	− 0.350	*	− 0.619	,	− 0.081
Motorcyclists that consumed alcohol	−0.221	*	−0.441	,	−0.001	− 0.106		− 0.286	,	0.073
Used illicit drugs	0.088		−0.210	,	0.386					
Used cellphone	−0.253		−0.615	,	0.108					
Use of safety devices	−0.111	**	−0.192	,	−0.029					
Work related	−0.156	**	− 0.249	,	−0.063					
Occurred on Sunday	0.172	***	0.079	,	0.265	0.088	*	0.013	,	0.163
Occurred during Daylight (07–19 h.)	− 0.269	***	−0.342	,	−0.197	− 0.077	*	− 0.138	,	− 0.016
Patient recruited in León	−0.423	***	−0.497	,	−0.348	− 0.391	***	− 0.458	,	− 0.324
Event occurred in a different municipality	0.417	***	0.323	,	0.512	0.076		−0.003	,	0.156
Municipality of occurrence not specified	0.334	***	0.211	,	0.458	0.111	*	0.011	,	0.212
Arrived to ER in a public/private service	0.839	***	0.778	,	0.900	0.767	***	0.707	,	0.826
Means of transportation to ER not specified	0.420		−0.021	,	0.861	0.396		−0.018	,	0.810
31–60 min before receiving medical attention	−0.375	***	−0.463	,	−0.288					
More than 60 min before receiving medical attention	− 0.443	***	−0.523	,	−0.364					
Time before receiving medical attention not specified	0.097		−0.140	,	0.334					

*p-value< 0.05; **p-value< 0.01; ***p-value< 0.001

^a^ Model fit: Log-likelihood: − 7439.6659; Likelihood Ratio Chi-square: 977.05; prob.>Chi-square: 0.0000; Pseudo R2: 0.0616; lnalpha: -1.04 (95% CI: −1.15, −.94); alpha: 0.35 (95% CI: 0.32, 0.39); Likelihood-ratio test of alpha = 0: Chibar2(01) = 2113.69, Prob≥chibar2 = 0.000; AIC: 14933.33

It should be noted that the use of drugs, cellphone use, use of safety devices, and whether the event was work-related lost statistical significance after controlling for the effect of other variables of interest and thus were excluded from the final model. In addition, no other interactions showed statistical significance in our sampled data (results not presented). Finally, the estimated alpha and the likelihood-ratio chi-square test provided evidence indicating that the negative binomial model was a better choice than a Poisson model (*p*-value = 0.000).

**Fig. 1:**
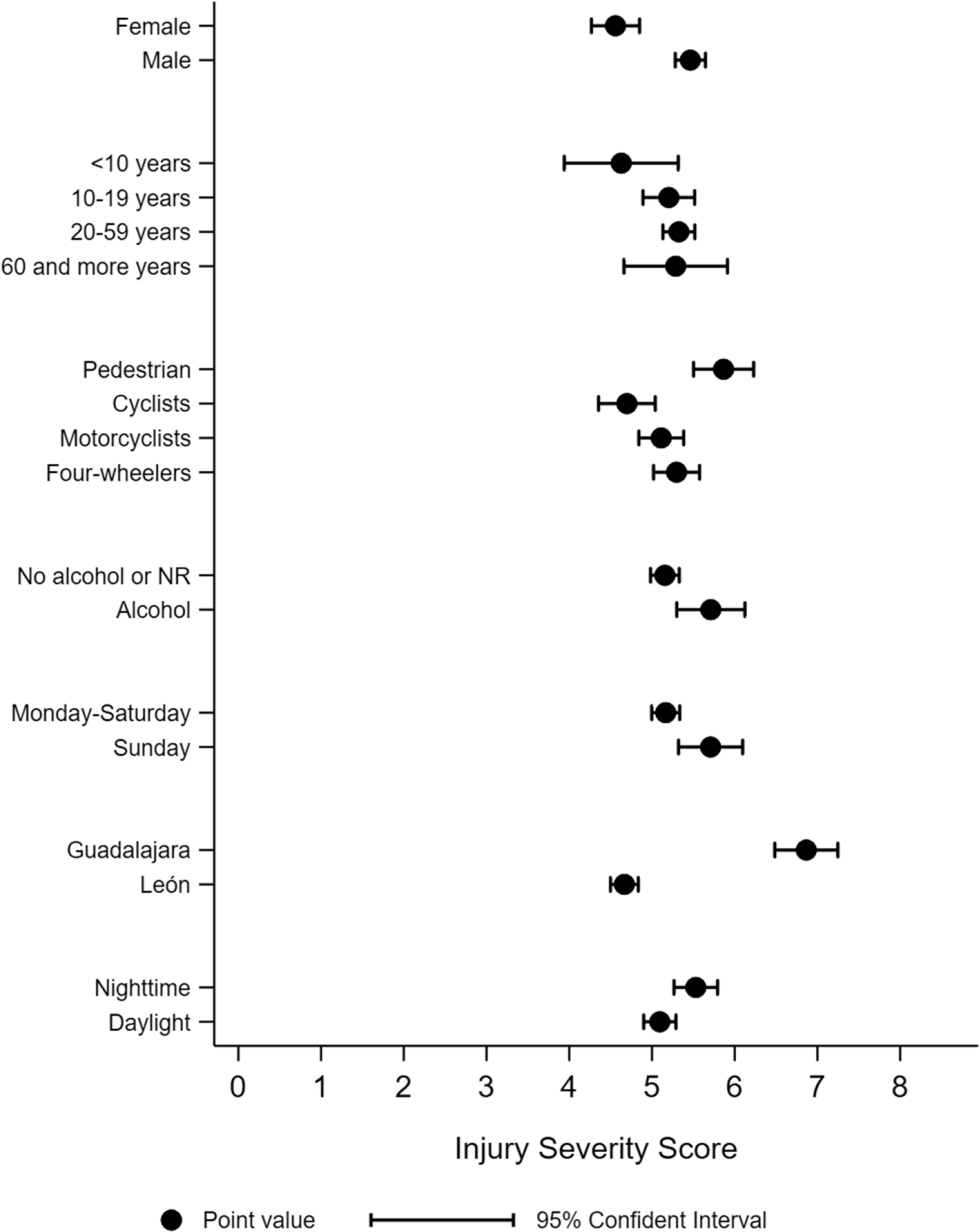
Estimated Injury Severity Score in road traffic injured from León and Guadalajara, Mexico

Figure 1 shows the expected ISS was higher for males (ISS = 5.5, 95% CI: 5.3, 5.6), pedestrians (ISS = 5.9, 95% CI: 5.5, 6.2), those consuming alcohol during the six hours before the event (ISS = 5.7, 95% CI: 5.3, 6.1), those events that occurred on Sunday (ISS = 5.6, 95% CI: 5.3, 6.0) and during nighttime (ISS = 5.5, 95% CI: 5.2, 5.8). The higher expected ISS was those injured persons recruited in Guadalajara (ISS = 6.9, 95% CI: 6.5, 7.3).

## Discussion

To our knowledge, this is the first study that analyzes the severity of multiple injuries sustained in road traffic collisions in Mexico and that identifies variables associated with it. A unique contribution of this work was the analysis of ISS in its natural state–that is, taking into consideration its inherent properties–using a Zero-Truncated Negative Binomial regression model. This approach had not been used before in the analysis of ISS showing good adjustment to our data. Previous studies have analyzed ISS as a dichotomous variable using a specific severity threshold (i.e. ISS of 8) [25]; as a categorical multinomial or ordinal variable of three-five categories [26–28]; or as a log-transformed continuous variable employing a general linear model [29]. Using our approach, the results allowed us to identify that the most severe ISS were registered among males, aged 20–59, with less schooling and whose traffic collisions occurred at nighttime; associations that have been extensively reported in previous studies. However, the use of illicit drugs, use of cellphones during the event and use of safety devices did not show association with ISS in our data. Besides, the results of this study have the potential to improve prevention strategies and in-hospital traffic injury care [30–32].

RTIs are commonly analyzed as if they were only one problem, while risk and protective factors, as well as preventive interventions, are different for each road user, highlighting the need to analyze them separately [33]. Supporting this argument, we documented a differentiated effect of alcohol among different road users. Although the interaction of these two variables has not been reported previously, there is clear evidence on the effect of alcohol consumption on the severity of injuries for different road users [34, 35]. Stübig et al. [28] and Pai et al. [36] found that there was a significantly higher speed at impact in injured patients with BAC positive as compared to those BAC negative (*p* < 0.0001). The interaction between alcohol consumption and type of road users allowed us to document that, in the absence of alcohol consumption; pedestrians had higher ISS than occupants of four-wheeled and other vehicles highlighting their vulnerability. Similar to our results, a study based on the trauma register in Finland found that pedestrians sustained more severe injuries than other road users [37]. In Poland, a study based on forensic data found that most fatal pedestrian victims sustained critical injuries, with a high percentage of deaths on the scene [38]. Even though alcohol was not significantly associated with severe injuries, age was a risk factor for critical harm in that same context [39]. This supports previous evidence showing that pedestrians might account for 45–53% of the overall mortality in Mexico [40, 41]. This is important to consider as it has been reported that most interventions to reduce RTIs, as well as research, are focused on motor vehicle occupants and little attention has been directed to other road users [42]. Pedestrian bridges are the most common intervention implemented in Mexico to prevent pedestrian crashes; studies demonstrate that they have been ineffective, besides generating other social issues, such as the assault on the bridges [43]. To prevent pedestrian injuries, a broader intervention is needed, making special adjustments to the road environment as well as enforcing comprehensive legislation aimed at reducing vehicle speed and increasing pedestrian visibility [42].

The fact that all cyclists and pedestrians that consumed alcohol had lower ISS than four-wheeled and other vehicle occupants that did not drink alcohol in our study could be the result of a higher lethality of traffic collisions for these users, i.e. that they tend to die on-site due to higher severity of their injuries. Stübig et al. [28] not only found that road users with alcohol intoxication had higher preclinical mortality but also, that BAC positive pedestrians, bicyclists and occupants of four-wheeled and other vehicles had higher ISS in comparison to BAC negative patients. This might have underestimated the specific effect of alcohol on vulnerable road users who represented more than 70% of our sample. However, the effect of alcohol on ISS was more evident in four-wheeled and other vehicle occupants who tend to survive more often because of the protection that the structure of the car or other vehicle itself provides. This highlights the need to prevent alcohol-related RTIs in four-wheeled and other vehicle occupants. Evidence indicates that the establishment of a legal breath alcohol concentration (BrAC) is a basic element to objectively detect alcohol impairment among drivers and enforce the law, [44] while random breath testing reduces the chance of missing impaired drivers [45]. Changes in the legislation were made during the study period: in Guadalajara, the legal BrAC was reduced in September 2013 and drink driving was considered a primary offense in both cities. In Guadalajara, this change took effect in August 2013 whereas in León went into effect in October 2012. In order to dissuade drivers, sobriety checkpoints might be combined with well-planned social marketing campaigns; conducted frequently, and have high public visibility [46, 47]. Despite being a cost-effective solution, we expect that the effect, in terms of injuries avoided, may increase with time and sustained deterrence actions [48].

The fact that the injured that arrived at the study hospitals by public/private ambulance services had more severe injuries could be thought of as a positive result in terms of the coverage of emergency medical services. However, from our study, it was still evident that at least 97 individuals with ISS of 9 or more arrived at the hospital by their own means, which is a cutoff point to consider ISS as serious [49]. This should not be happening if a better prognosis is expected for serious RTIs. In addition, timely and high-quality medical care is important. As documented in a parallel study, the time before receiving medical attention was not associated with the final health outcome of the injured [50]. This evidence supports the need for improving effective and opportune coverage of EMS particularly in these two cities, but in Mexico in general.

Collisions occurring in a different municipality than where both hospitals are based apparently translated to more severe injuries. This might be explained by the fact that both hospitals are referral centers and thus patients coming from less developed medical care centers from surrounding municipalities tend to be the most severe. In this sense, patients in Guadalajara had more severe injuries as well, presumably because the Civil Hospital Fray Antonio Alcalde is a larger and more developed medical center. Another explanation could be that events might have occurred in inter-city roads where the circulation of motor vehicles tends to be at higher speeds, producing more severe injuries. This could be supported by other findings of our study showing that collisions occurring on Sundays and at nighttime produced higher levels of ISS, as less congestion allows motor vehicles to exceed speed limits [51]. A study carried out in Kenya also found that nighttime was associated with the severity of RTIs [52].

Lack of statistical association between the use of safety devices and ISS might be counterintuitive. However, this might be due to selection bias, as those with higher severity of injuries tend to die on-site and thus were not included in our analysis. Future studies should gather information from those who died before arriving to the hospital and those who required only prehospital care and did not receive hospital attention for a more integral analysis. Although this may be an important limitation of our study, it could also suggest a lower use of safety devices (and possible exposure to other risky behaviors) among those sustaining RTIs as compared to the general population. In addition, helmet use in motorcyclists was documented to be between 62.7 and 85.9% in the general population of the same two cities during the same period of time [53] compared to 44.7% observed in our study sample. Likewise, a previous study documented the use of restraint devices at 39.6% in all four-wheel occupants [54] while in our analysis it was only 29.7%. It could also mean that our sample included people with lower socioeconomic status for the most part as they tend to be mainly attended in public hospitals. Previous evidence shows that the use of safety devices tends to be higher in individuals with higher education [27, 55, 56].

Considering the convenience sampling of hospitals, our results may not be generalizable to the larger population. Besides, our sample size might be small to demonstrate exposure to unusual events such as drug use or cellphone use during the collision. The low prevalence of those events may be influenced by the method used (i.e. self-report) as individuals are prone to report lesser use due to social desirability. Evidence from two observational studies reported a higher prevalence of cellphone use in the same context [57, 58].

Due to the data collection method, we could not gather information from those who required only pre-hospital care and did not require hospital attention or those who died before arriving to the hospital, which is a limitation in terms of having the complete picture of what happens to the injured before receiving any kind of care. To cover all the events, in the future, it is necessary to implement broader injury surveillance, and to include pre-hospital care information. Despite the limitations of our study, we believe our results provide valuable information to local and national decision-makers.

## Conclusion

These findings support the urgent need to implement comprehensive and multi-sectorial road safety interventions and programs mainly focusing on vulnerable road users, motorcyclists were the most affected road user in both cities and pedestrians are the road user with most severe injuries. Alcohol consumption was significantly associated with a higher severity of injuries, 14.9% of those injured in a traffic collision, self-reported alcohol consumption.

Prioritizing events occurring on Sundays at night seems to be a feasible way of decreasing the severity of multiple injuries sustained in these two cities. Emphasis must be set on road safety legislation and its appropriate enforcement, to improve road infrastructure considering the requirements of vulnerable road users, and to improve the emergency medical systems’ response [16]. Improving pre-hospital and emergency care may not only contribute to the reduction of the burden of RTIs but also improve attention to other trauma and acute medical conditions. Previous studies had estimated that such enhancement might prevent 34–38% of injury-related deaths in LMICs [59]. Serious injuries were observed in 15.4% of the cases while critical injuries in almost 5%.

## Data Availability

The datasets used and /or analyzed during the current study are available from the corresponding author on reasonable request.

## Abbreviations

AIC: Akaike’s Information Criteria
AIS: Abbreviated Injury Score
BAC: Blood alcohol concentration
BrAC: Breath alcohol concentration
CI: Confidence interval
ER: Emergency rooms
HIC: High-income countries
ICD: International Statistical Classification of Diseases and Related Health Problems
ISS: Injury severity score
LMIC: Low-and middle-income countries
RTI: Road traffic injuries
SD: Standard deviation
